# Carrot and stick: how RNase R contributes to function and destruction of the translation machinery

**DOI:** 10.1080/15476286.2025.2535846

**Published:** 2025-07-29

**Authors:** Helge Paternoga, Lyudmila Dimitrova-Paternoga

**Affiliations:** Institute for Biochemistry and Molecular Biology, University of Hamburg, Hamburg, Germany

**Keywords:** RNase, RNase R, RNA, ribosome, RNA decay, RNA degradation, RNA turnover, bacteria, translation, structure

## Abstract

RNA is fundamental for life, and its homoeostasis is a critical contributor to cellular growth and adaptation to stress. Key RNA species include messenger RNA (mRNA) and non-coding RNAs, such as transfer RNA (tRNA), or ribosomal RNA (rRNA), that are essential for ribosome formation and translation of the genetic code. Furthermore, various other non-coding RNAs are expressed at each growth stage. Given RNA’s abundance and its role in all cellular processes, RNases – enzymes responsible for RNA degradation and processing – are central to RNA metabolism. In this review, we discuss the pivotal contribution of the 3’ exonuclease RNase R to bacterial RNA homoeostasis. We focus on its functions in regulating and degrading components of the translation machinery, including the trans-translation system, and we take a look at recent structural studies that shed new light on the activities of this important enzyme.

## Introduction

Most organisms express multiple RNases, which often display functional redundancy and can work in concert within various RNA degradation and processing pathways. These enzymes are broadly classified into endoribonucleases, which cleave internal phosphodiester bonds, and exoribonucleases, which degrade RNA from either the 5’ or 3’ end. The presence and activity of specific RNases in an organism shape how RNA levels are regulated. For instance, the gamma-proteobacterium *Escherichia coli* possesses more than 20 distinct RNases, about half of which are absent in distantly related bacterial species, like *Bacillus subtilis* [1]. Nevertheless, a core set of RNases is conserved across nearly all bacteria (reviewed in detail in ref. 1).

This review focuses on one important bacterial core RNase, called RNase R, which plays a key role in stress survival and pathogenicity of many bacteria [2]. RNase R is a 3’ exonuclease that belongs to the universally conserved RNB protein family (named after the RNB domain that is unique to this protein family) [3]. It acts on all classes of RNA substrates in the cell, including housekeeping RNAs, namely tRNA, mRNA, rRNA, as well as small non-coding RNAs, like 6S RNA [4]. Furthermore, RNase R has been implicated in boosting type III CRISPR-Cas immunity (Clustered regularly interspaced short palindromic repeats) in *Staphylococcus epidermidis* through the processing of crRNAs (CRISPR RNA) [5], as well as an interactor of a phage-encoded anti-CRISPR protein purified from the same organism [6]. Another interesting aspect is that it protects *Pseudomonas syringae* from oxidative and DNA damage and is required for growth at low temperature in this psychrotrophic bacterium [7,8].

RNase R is a major 3’ exonuclease in bacteria together with its family member RNase II and polynucleotide phosphorylase (PNPase). All three enzymes are processive and act in a partially redundant manner. In *Escherichia coli*, double deletion of RNase R with PNPase is lethal, underscoring the high importance of this enzyme [3]. As an important regulator of RNA homoeostasis, RNase R contributes to the virulence of many bacteria (reviewed recently in ref. 2) and it is being actively studied as a target for the development of novel antimicrobial compounds [9].

RNase R is a unique exonuclease capable of unwinding even highly structured RNA substrates, allowing it to degrade nearly any RNA molecule with a short single-stranded 3’ overhang [10]. This ability is harnessed in the study of circular RNAs, as these do not contain 3’ overhangs and are thus resistant to RNase R treatment, enabling their enrichment upon RNase R digestion of RNA mixture samples [11–13].

In this review, we will focus on important functions of RNase R in regulating the translation machinery and ribosome turnover which are informed by recent insights into the structure and function of this unique RNA decay enzyme.

## RNase R belongs to the RNase II/RNB family of proteins

The RNase II/RNB family of ribonucleases includes major nucleases in all domains of life. Most bacteria possess two homologs, RNase R and RNase II [3], which are also present in archaea [14] and bacteria-derived organelles, like chloroplasts [15]. In eukaryotes further cytoplasmic and nuclear paralogs exist, a key example is Rrp44/Dis3 in *Saccharomyces cerevisiae* and humans, which serves as the catalytic subunit of the exosome complex [16]. Humans even possess three Dis3 homologs: Dis3, which associates with the nuclear exosome, Dis3L, which associates with the cytoplasmic exosome and Dis3L2 which functions independently from the exosome [16].

**Figure 1.**
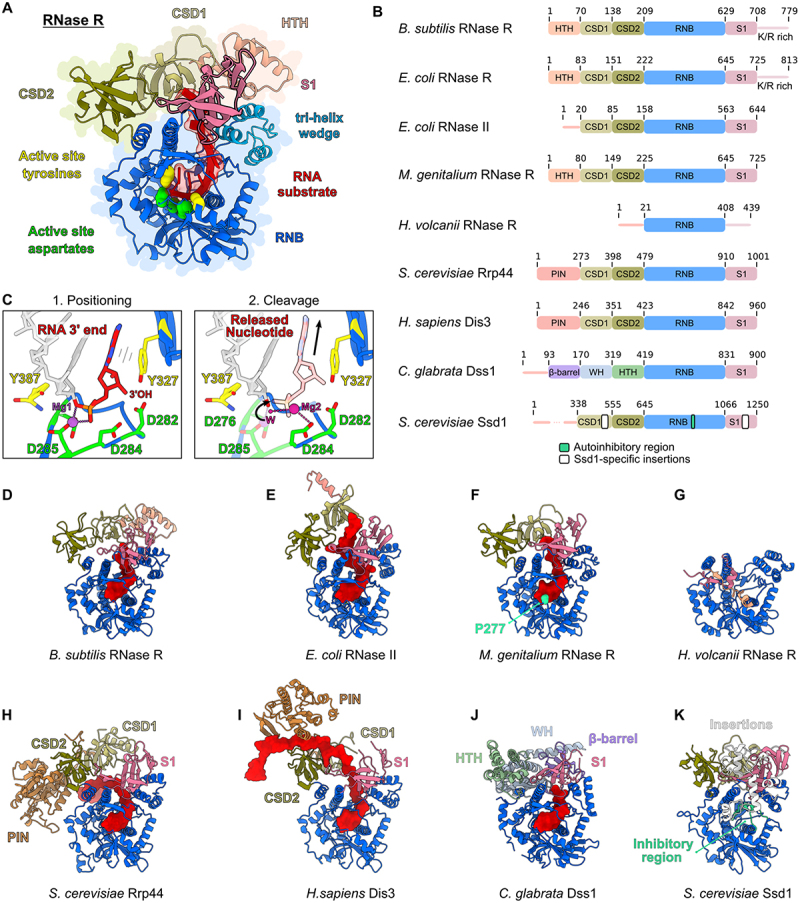
Structures of RNase R and its homologs: (A) *M.genitalium* RNase R (PDB: 7DIC) with the HTH domain superposed from the *B.subtilis* protein (PDB: 8CDU). The N-terminal HTH domain (pink) is followed by two cold shock domains (CSD1 (light green) and CSD2 (dark green), RNB domain (blue) and a S1 domain (beige). The active centre aspartates are depicted in green and the tyrosines which hold the substrate in place are depicted in yellow. The tri-helix wedge of the RNB domain is coloured in light blue. (B) Domain organization of RNase R homologs from different organisms, the molecular models of which are presented in panels (D-K). The S1 domain of RNase R is followed by a K/R-rich extension. RNase II is missing the N-terminal HTH domain. *H.volcanii* RNase R presents only rudimentary accessory domains. (C) Left panel: active centre of RNase R (PDB: 7DIC) with the RNA substrate shown in red, one Mg^2+^ ion in purple, active centre aspartates in green and the tyrosines which hold the substrate in yellow. The right panel depicts the position of the second Mg^2+^ ion and is superimposed from the structure of *Trypanosoma brucei* Rrp44 (PDB: 7TUV) on the MgRNase R structure. (D-K) Atomic models of D) BsRNaseR (PDB: 8CDU). (E) EcRNase II (PDB: 2ID0). (F) MgRNaseR (PDB: 7DIC), P277 that confers methylation-sensitivity is shown in cyan. (G) HvRNase R (AlphaFold model^25^). (H) ScRrp44 (PDB: 4IFD). (I) HsDis3 (PDB: 6D6Q). (J) CgDss1 (PDB: 6F3H). (K) ScSsd1 (PDB: 7AM1), Ssd1 is an inactive RNase R homolog with an inhibitory alpha helix that blocks substrate binding (blue-green).

Members of the RNB family share a common domain architecture centred around a catalytic RNB domain (Figure 1(A)). This domain is flanked in most members by two cold-shock domains (CSDs) at the N-terminus and a ribosomal protein S1 domain (S1) at the C-terminus (Figure 1(A,B)). In contrast to RNase II, RNase R possesses an additional helix-turn-helix (HTH) domain N-terminally to CSD1 and a C-terminal lysine-arginine rich (K/R-rich) unstructured region. Unlike other family members, Rrp44, Dis3 and Dis3L contain an N-terminal endonuclease PIN domain, that also mediates the interaction with the exosome (Figure 1(B)) [16].

It has been shown that the mechanism of catalysis is conserved in the RNB family and that it utilizes two metal ions that are coordinated by key conserved aspartic acid residues in the RNB domain (Figure 1(C)) [17–20]. In addition, through structural analysis it has become clear that the three-dimensional arrangement of shared domains is highly conserved as well (Figure 1(D–K)), nevertheless important functional differences exist between family members:

Although RNase R and RNase II share a highly similar domain architecture (Figure 1(B,D,E)), their substrate preferences differ. RNase R is capable of degrading structured RNAs, whereas RNase II is only able to digest single-stranded RNA substrates that do no possess extensive secondary structure [21]. Furthermore, family members that deviate from the consensus blueprint exist. Examples include the mitochondrial RNase Dss1, which contains a unique set of accessory domains in front of the RNB domain, or the Ssd1 protein which has lost its RNase activity altogether and instead functions as a translational regulator (Figure 1(B,J,K)) [22,23]. In addition, RNB family members exist, that consist mostly of an RNB domain, with minimal accessory domains. Examples include *Deinococcus radiodurans* RNase II [24] and *Haloferax volcanii* RNase R [14,25] (Figure 1(B,G)). Whereas the *D. radiodurans* homolog has been identified as RNase II [24], the *Haloferax* homolog shows a remarkable temperature-dependent ability to switch from an RNase II-like behaviour at lower temperature to an RNase R-like behaviour at optimal growth temperature, which includes the degradation of structured RNA substrates that is a hallmark of RNase R activity [14].

## Mechanism of action of RNase R

The RNB domain of RNase R, which is the unique characteristic of the RNB family of proteins, is highly similar to that of RNase II and scRrp44/Dis3 (Figure 1(D,E,H)). It features a unique αβ-fold with a central lumen that can accommodate around 10 nucleotides of ssRNA [17,26]. The bases in the RNase lumen are packed together in a parallel stacking conformation with the phosphate groups involved in hydrogen bonding and salt bridges with the surrounding amino acids [18,26]. This family of ribonucleases employs a two-metal-ion catalytic mechanism, similar to that of RNase H [17]. In the case of *Mycoplasma genitalium* RNase R, a single magnesium ion (Mg^2+^) is coordinated by the conserved aspartates 276 and 285 (D276 and D285) (Figure 1(C)). These are equivalent to D201 and D210 in *E. coli* RNase II [17]. Although most of the available structures of the RNB domain feature only one bound magnesium ion, a second magnesium ion is hypothesized to coordinate a water molecule. In this model, two additional aspartates function as a general base, deprotonating the Mg^2+^ -coordinated water, which then facilitates a nucleophilic attack on the phosphodiester bond of the RNA substrate, enabling its cleavage (Figure 1(C)) [17,19,20]. Notably, a recent structure of *Trypanosoma brucei* Rrp44 revealed, for the first time, the presence of a second magnesium ion modelled at D284 (using *M. genitalium* numbering; Figure 1(C)) [18]. Its position appears to be influenced by the catalytic state of the enzyme, potentially explaining why this second magnesium is absent in other structures that are based on catalytically inactive RNase R mutants. Interestingly, this structure appears to present a post-catalysis state of the enzyme, as the nucleotide in the active centre is split off the rest of the substrate [18]. Additionally, two tyrosines Y327 (Y253 in *E. coli* RNase II) and Y387 (Y313 in *E. coli* RNase II) clamp the substrate at the active centre by stacking interactions. In the post-catalysis state, the cleaved most 3’ terminal nucleotide retains the stacking interaction with the conserved Y327 (Y555 in *T. brucei*), whereas its phosphate group undergoes structural rearrangements [18] (Figure 1(C)). A further important residue that stabilizes the substrate in the 5’ direction is L432 (L656 in *T. brucei*, F358 in *E. coli* RNase II) [17–19].

Although RNase R is a robust RNase that can unfold even structured RNA, recent work has highlighted that the enzyme is sensitive to cytosine-rich stretches which inhibit its activity [27]. Furthermore, RNase R of *M. genitalium* shows a remarkable sensitivity to substrate modification. Multiple groups have shown that *M. genitalium* RNase R (and also some other RNases from the *Mycoplasmatacea* family) is inhibited by 2’-O-ribose methylation [19,28,29]. Structural and biochemical analysis suggests that proline 277 of *M. genitalium* RNase R and an adjacent loop affect the sensitivity of this enzyme to 2’-O-ribose methylation. It is assumed that this proline residue slows down the translocation of the substrate during hydrolysis [19,29] (Figure 1(F)). 2’-O-ribose methylation plays an essential role in the regulation of gene expression and is implicated in human diseases such as cancer or autoimmune conditions [30]. Therefore, it has been suggested that the sensitivity of MgRNase R could be harnessed in the future as a tool to analyse the methylation state of RNA [19].

## How can RNase R degrade structured RNA?

A defining function of RNase R is its ability to degrade highly structured RNA substrates, which sets it apart from its family member RNase II. Although the general domain architecture is shared between these two proteins, it has been shown that the lone RNB domain of RNase R is sufficient to confer substrate unwinding, albeit with reduced efficiency compared to the full-length protein [14,31–33]. Additionally, it has been demonstrated using single-molecule fluorescence analysis that Rrp44, a close paralog of RNase R, utilizes the energy from RNA-hydrolysis to directly unwind structured substrates [34]. It is likely that RNase R uses the same strategy to unfold substrates, as the active site of the RNB family (Figure 1(A,C)) is universally conserved [18,19]. Furthermore, a previous notion that RNase R contains an ATP-dependent helicase activity [35] could not be confirmed by later work [26]. Therefore, it appears that RNA substrate binding and hydrolysis are the main steps that regulate the unwinding force of RNase R. In this light, it is highly interesting that the RNB domain of RNase R binds RNA more tightly than the equivalent domain of RNase II [33], which can explain the differences in substrate specificity and catalysis rate between these two proteins [33]. Because RNase R can interact more tightly with RNA, it has been proposed that it can utilize the natural molecular breathing of RNA molecules in solution (the dynamic and rapid breakage and reformation of base-pairs) to drive unwinding [32]. In this ‘thermal breathing model’ RNase R advances on partially opened RNA-duplexes and wedges them open to feed unfolded single-strand RNA into its active centre [32].

Several studies have shed light on the contribution of individual domains of RNase R towards substrate unwinding: One part of the RNB domain which has been designated as a ‘tri-helix wedge’ has been shown to be especially important [26,36]. The tri-helix wedge is located at an exposed position at the top of the RNB domain and is directly adjacent to the substrate path towards the active site (Figure 1(A)). Furthermore, the tri-helix wedge sits in close proximity to the S1 domain (Figure 1(A)) that has also been shown to contribute to substrate unwinding activity in full-length RNase R. By studying a hybrid protein containing the RNB domain of RNase II, but the S1 domain with its K/R-rich extension of RNase R, Matos and co-workers have demonstrated that these domains can grant the hybrid enzyme the ability to degrade double-stranded substrates [21]. Furthermore, conserved residues in the S1 domain together with the K/R-rich extension are important for binding of duplex RNA [31,33]. This suggests that accessory domains at the RNB domain can contribute to unwinding activity by regulating RNA binding surfaces *in vivo* [26].

## CSD domain flexibility contributes to substrate turnover

A large focus in the past has been placed on defining the entryway of RNA substrates towards the active site of the enzyme. Through structural analysis, two substrate paths have been described, the ‘top’ and ‘side’ channels that reflect how substrates can enter the enzyme relative to the active site [19,26]. For example, the side channel is prominently observed in structures of yeast Rrp44-exosome complexes (Figure 1(H)), whereas the human homolog Dis3 bound to the exosome shows substrate entering through the top channel (Figure 1(I)). These analyses are based for the largest part on crystal structures of RNase R or other members of the RNB family in complex with various RNA substrates. In these studies the positioning of the cold-shock domains is crucial and defines openings that allow or block RNA substrates from entering [17]. CSD domains are conserved throughout all kingdoms of life and consist of a five-stranded anti-parallel beta barrel, capped by an alpha helix. Despite a low degree of sequence conservation between the various CSDs, these domains display a conserved DNA/RNA binding surface [37]. It has been discussed that the CSDs in RNase R serve as an initial barrier of duplex-RNA binding to the RNB domain [33,38]. It has been noted that the general position of the CSDs between homologs is conserved, but may vary relative to the nuclease domain, which has resulted in the definition of the two substrate channels mentioned above.

**Figure 2.**
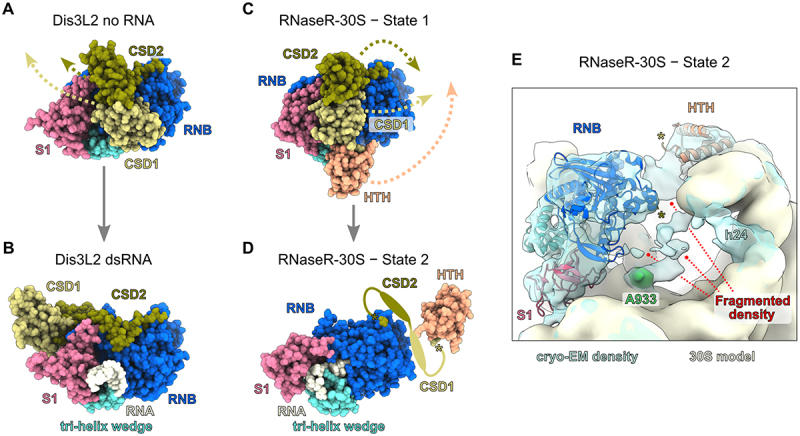
The cold-shock domains of RNB family members are flexible. A) RNA-free state of human Dis3L2 (PDB: 8E27). B) Human Dis3L2 bound to the D-U7 hairpin (PDB: 8E2A). The CSDs have moved by ~ 70Å allowing the double-stranded portion of the RNA-duplex to access the tri-helix wedge. C) State 1 of *B.subtilis* RNase R bound to a degradation intermediate of the small ribosomal subunit (PDB: 8CDU). D) State 2 of *B.subtilis* RNase R bound to a degradation intermediate of the small ribosomal subunit (PDB: 8CDV). CSD repositioning is inferred from the position of the adjacent HTH-domain. The CSD domains could not be modelled due to flexibility in this state. E) cryo-EM density of state 2 of *B.subtilis* RNase R bound to a degradation intermediate of the small ribosomal subunit. The filtered cryo-EM density is shown in transparent blue (EMD identifier 16596). The molecular model of the 30S subunit is shown in beige. Fragmented density at the neck region of the small subunit is indicated by red lines. The S1 domain of RNase R is not part of the molecular model (PDB: 8CDV) and has been rigid body fitted in the cryo-EM density (using PDB entry 8CDU).

The definition of substrate channels is based predominantly on crystal structures, that show the most stable ‘frozen’ state of RNase R in complex with its substrate. Therefore, more dynamic or flexible conformations of the accessory domains remained elusive. With recent structural work, it has become apparent that the cold-shock domains of RNB family members are much more flexible and dynamic than previously shown [19,36,39]. First, Abula and colleagues have noted that the certainties for assigning the CSDs in their crystal structures were very low, when a double-stranded RNA substrate was incubated with RNase R, compared to the apo-form or when using a single-stranded RNA [19]. This suggests that double-stranded RNA can induce molecular movements in the cold-shock domains, hampering their structural analysis by crystallization. In a recent elegant study, using cryogenic electronic microscopy (cryo-EM), Meze and co-workers showed, that the CSDs of the RNB family nuclease Dis3-like 2 (Dis3L2) display a very similar behaviour to RNase R when the enzyme is incubated with double-stranded RNA [36]. By resolving multiple structures of Dis3L2 in complex with various RNA hairpins Meze et al. demonstrated that the CSDs reposition dramatically, by over 70Å, once structured RNA binds to the enzyme [36] (Figure 2(A,B)). Through the study of a ΔCSD mutant it has become clear that the CSDs contribute to both the initiation of RNA degradation and to substrate association during the initial unwinding steps [36]. Subsequently, when the CSDs have moved away, one side of the RNA duplex is positioned above the tri-helix wedge at the RNB domain (Figures 1(A) and 2(B)), which is highly important for unwinding of structured substrates [26,36]. Through their work, Meze et al. define 6 crucial steps, by which RNB family members unwind and degrade structured RNA: First, substrate associates with the nuclease (1) and initial nucleotide cleavage occurs (2). Then, the single-stranded part of the substrate is degraded (3) until a double-stranded region engages the enzyme (4). This is followed by a dramatic domain rearrangement (5) and subsequent unwinding and degradation of the structured RNA (6). Importantly, the work of Meze et al. suggests that most members of the RNB family would have to undergo a similar conformational change to allow the access of a double-stranded RNA to the tri-helix wedge [36].

This assumption is supported by a recent cryo-EM study of RNase R, which has been carried out using native pullouts of the enzyme [39]. In this work, we showed that RNase R co-purifies degradation intermediates of small ribosomal subunits (SSU) and we identified two cryo-EM states that capture different steps of SSU turnover (see section on rRNA turnover below). The first state shows the accessory domains in a similar ‘default’ conformation to the one observed for other RNB family members (Figure 2(C)). Importantly, the second state revealed a dramatic rearrangement of the N-terminal HTH domain, which is directly connected to the cold-shock domains (Figure 2(C,D)). Although, the cold-shock domains themselves could not be resolved, we observe a fragmented cryo-EM density at their expected location (Figure 2(E)). Therefore, it can be inferred that the CSDs are moved away to the opposite side of the RNB domain, compared to the structures of Dis3L2 (Figure 2). This position of the CSDs not only opens up the access to the tri-helix wedge (shown on the left side of Figure 2(E)), but could also assist in destabilizing the ribosomal RNA in this region (see section on rRNA turnover below).

It has therefore become apparent that the simple classification into ‘top’ and ‘side’ substrate channels does not cover the full picture of substrate feeding into the active site of RNB family exonucleases. Therefore, it will be crucial to understand how other members of the RNB family employ rearrangements of their accessory domains to process highly structured RNA substrates.

## RNase R – a sentinel of ribosome activity

RNase R is central to life and contributes to a wide range of RNA decay functions, mirroring the importance of its RNA targets. The association with translation components stands out as a central theme for RNase R and much work has been dedicated to elucidate its contribution to ribosome-related processes. In most cases, RNase R has been shown to adopt a sentinel function which removes aberrant or excess RNA molecules from the cell, but a few studies indicate additional contributions to RNA processing. Below we discuss how RNase R participates in the regulation of translation components, ribosome quality-control, as well as ribosome homoeostasis.

## tRNA maturation and quality control

There are only a handful of studies that analysed the contribution of RNase R to tRNA maturation, a process that typically requires multiple RNases in most bacteria [40,41]. For example, in the pathogenic bacterium *M. genitalium*, where RNase R appears to be the only 3’ exoribonuclease, this enzyme processes tRNA 3’ ends in a one-step mechanism [19,28,40,42]. A similar contribution to tRNA maturation has not been observed in other bacteria so far, although only a limited subset of tRNAs have been analysed to date in *E. coli* [43] or *B. subtilis* [44–46]. Therefore, it remains to be seen if the processing mechanism of *M. genitalium* has specifically evolved only in this genome-reduced organism.

RNase R however appears to have a broad function in the turnover of defective or truncated tRNAs, which is observed in *E. coli* and *B. subtilis* [44,45,47,48]. Because RNase R requires a single-stranded overhang of ~ 7 nucleotides to effectively engage structured substrates, the addition of such overhangs to the 3’ ends of tRNAs, either through poly-adenylation [41,44,49,50] or by the CCA-adding enzyme [44,47,48], contributes to RNase R-mediated decay [44,47]. It remains to be explored if other systems that can extend tRNAs with 3’ overhangs, like the MenT toxins [51–53], can tag tRNAs for degradation by RNase R.

Interestingly, a different toxin family has been discovered by the Hauryliuk and Atkinson labs that acts by adding pyrophosphate to the 3’ end of tRNAs [54]. Such a system could inhibit RNase R activity on tRNAs, as pyrophosphate added to the 3’OH of a ribose-ring might not properly fit into the active site of the enzyme (Figure 1(C)).

## mRNA turnover

mRNA degradation is an integral part of cellular homoeostasis, as the pool of mRNAs present in the cell directly determines which proteins are available to respond to environmental changes. Accordingly, mRNAs generally possess short half-lives, allowing a speedy adaptation to changes in growth conditions, or to cellular insults [55]. RNases therefore carry out the important function of removing mRNAs that are not needed anymore. RNase R can participate in mRNA degradation [56–59], and several studies have analysed the contribution of the enzyme to bulk mRNA turnover [55,60–65]. Furthermore, RNase R plays a pivotal role in the degradation of mRNAs that get targeted during trans-translation (see below) or mRNAs that become structured upon cold-shock [66]. In cells that are deleted for RNase R, the fraction of mRNA in the total cellular RNA increases from < 4% to 10% during an 8 hour long cold-shock treatment, which was not observed in wild type cells [66]. It has been shown that RNase R acts on structured mRNAs even before cold-shock proteins have been expressed [66]. Therefore, RNase R plays a crucial role in overcoming the initial down-regulation of translation that occurs after cold-shock so that adaptation to cold conditions can occur. One more specific cold-shock effect has been reported in *Streptococcus pneumoniae* where it has been demonstrated that the *smpB* mRNA, encoding for SmpB (an important component of the trans-translation system, see below), is targeted by RNase R under cold-shock conditions [67]. Apart from cold-shock, RNase R controls the expression levels of translation factors RRF, EF-G and IF3 in *Streptococcus pneumoniae* [68] and contributes to the turnover of *rpsO* mRNA in *E. coli* and *B. subtilis* [56,58]. *rpsO* encodes for the ribosomal protein uS15 and is situated in both organisms upstream of the *pnp* gene, albeit with one annotated transcription terminator in between [69,70]. *pnp* encodes for PNPase, an important bacterial 3’ exonuclease [71,72], that itself is involved in mRNA turnover [56,57]. It has been demonstrated in *B. subtilis* that RNase R degrades the *rpsO* mRNA when PNPase is deleted [56]. Similarly, in *E. coli* degradation of the *rpsO* mRNA by RNase R is observed, when RNase E is inactivated [58]. Furthermore, RNase R has been shown to target structured mRNAs, containing repetitive extragenic palindromic sequences (known as ‘REP’ sequences), together with PNPase [59]. Finally, the deletion of *rnr* (the gene encoding RNase R) in *E. coli* cells leads to the accumulation of *ompA* mRNA in stationary phase, indicating a specific contribution of RNase R to the expression level of single mRNAs [57].

## tmRNA and trans-translation

Once mRNAs are translated, they are generally protected from RNase digestion [56,73–76]. But when translation encounters a problem, ribosome-associated mRNAs are often degraded utilizing specialized quality control factors that work together with RNases. Furthermore, when faulty or truncated mRNAs are translated, ribosomes become stalled which causes traffic jams when following ribosomes run into the stalled one. This situation is highly detrimental to the cell because all collided ribosomes cease to produce proteins and because partial protein products from these complexes can be toxic. Stalled ribosomes do not reach the stop codon on the mRNA and therefore cannot be released by canonical translation factors and stay associated with the mRNA. To resolve such complexes, all living cells contain ribosome rescue systems that detect and repair translational stalls, in a process called ‘ribosome quality control’. Most organisms contain more than one rescue system, underscoring the high importance of resolving ribosomal stalls for the cell [77]. For example, bacteria can contain either the trans-translation system [78], the alternative rescue factors ArfA/BrfA [79,80] and ArfB [81], PrfH (protein release factor homolog) [82,83], or the ribosome-associated quality control system (RQC) [84].

**Figure 3.**
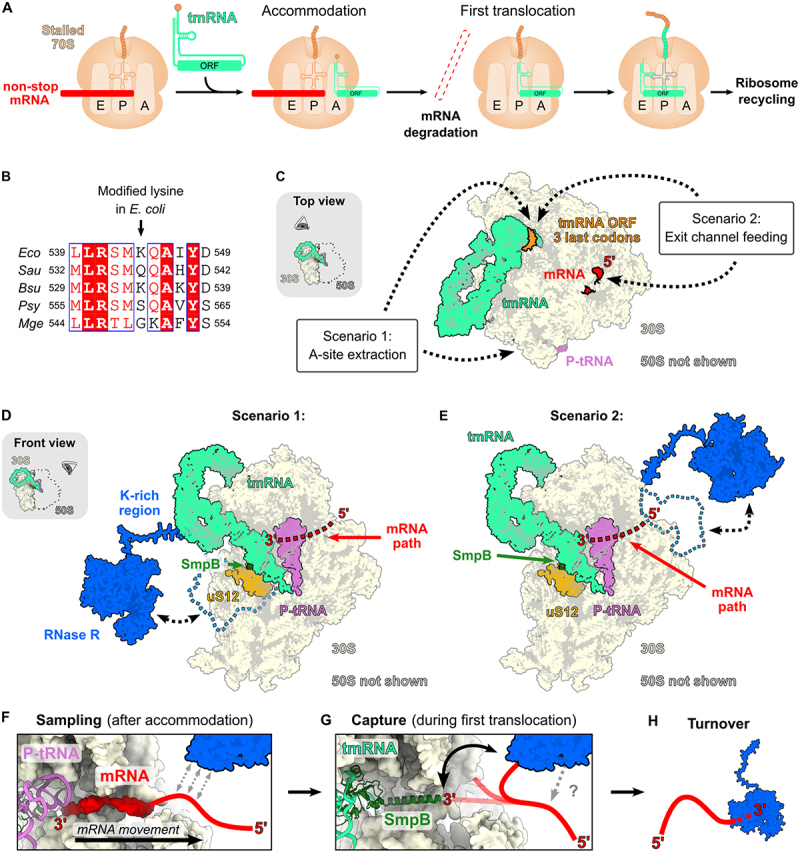
Model for the contribution of RNase R to non-stop mRNA degradation during trans-translation. (A) Schematic of the trans-translation reaction. A stalled ribosome containing non-stop mRNA is engaged by tmRNA that accommodates into the ribosomal A-site. After accommodation, the truncated mRNA gets released from the ribosome and is degraded. This activity is connected to the first round of translocation on tmRNA. Subsequently, the tmRNA ORF is translated, during which a degron tag is added to the nascent protein chain. This is followed by canonical termination of translation. (B) Sequence alignment of the region of lysine 544 in *E.coli* RNase R across multiple bacterial species. Eco = *E.coli*, Sau = *S.aureus*, Bsu = *B.subtilis*, psy = *P.syringae*, mge = *M.genitalium* . The alignment was prepared using ESPript (https://espript.ibcp.fr/ESPript/cgi-bin/ESPript.cgi). (C) Overview of two possible scenarios of RNase R engagement onto stalled 70S ribosomes. RNase R associates with tmRNA-bound ribosomes by recognizing conserved features of the tmRNA ORF sequence (orange). RNase *R* = blue, tmRNA = green, mRNA = red, P-tRNA = pink. 30S subunit = beige. The 50S subunit is not shown. The figure is based on PDB entry 7AC7. (D) Scenario 1 of RNase R function in tmRNA-mediated non-stop mRNA decay. The enzyme engages the 30S subunit from the A-site region and directly engages the 3’ end of the truncated mRNA in the P-site. E) Scenario 2 of RNase R function in tmRNA-mediated non-stop mRNA decay. RNase R binds at the mRNA exit channel and samples the mRNA through an RNA-binding interface. When the 3’ end of the mRNA is released from the ribosome, RNase R captures it and feeds it into its active centre. (F) Close-up of the state before the first round of tmRNA translocation. Sampling by RNase R is indicated by grey arrows. RNase R = Blue. mRNA = Red. P-tRNA = pink. Based on PDB entry 7AC7. (G) Capture of the mRNA during the first round of translocation. This could be supported by an additional interaction of the enzyme on the mRNA, holding it in place (indicated by the grey question mark and arrow). RNase R = blue, tmRNA = green, mRNA = red. Based on PDB entry 7ACJ. (H) Turnover of mRNA. RNase = blue, mRNA = red.

Trans-translation is the dominant rescue system in most bacteria and involves several steps (Figure 3(A)). It is carried out by transfer-messenger RNA (tmRNA) and its protein binding partner SmpB [78,85]. tmRNA is a special RNA molecule that contains both a tRNA-like fold, as well as an open reading frame (ORF) which importantly provides a valid stop codon. To rescue stalled 70S ribosomes tmRNA needs to become charged with alanine at its tRNA-like domain and to form a complex with its binding partner protein, SmpB [85–87]. The tmRNA-SmpB complex binds to the A-site of a stalled ribosome (Figure 3(A)), where SmpB ensures that the site is free of mRNA before binding occurs [88]. Importantly, truncated mRNAs lacking a stop codon (non-stop mRNAs) directly present an open A-site, as the ribosome translates to the very end of the mRNA before stalling, and is therefore directly accessible by the tmRNA machinery (Figure 3(A)). In the case that mRNA is present in the A-site, prior cleavage by an endonuclease is required to free the A-site for tmRNA-SmpB [89–93]. The list of known endonucleases that can cleave mRNA on the ribosome is still incomplete, but it is known that members of the RelE toxin family in *E. coli* and *M. tuberculosis* [94–99] as well as the endonuclease Rae1 in *B. subtilis* [100–103] can attack mRNAs in the ribosomal A-site. Furthermore, studies in *E. coli* have highlighted the involvement of further RNases that prepare ribosomal complexes for these cleavage events [93,104]. Once tmRNA is accommodated into the A-site, the alanine at the tRNA-like fold attacks the stalled nascent chain in the peptidyl transferase centre and attaches to it, effectively taking over the translation reaction [78]. Concomitantly, a first round of translocation occurs in which the tRNA-like domain is moved to the P-site of the stalled ribosome (Figure 3(A), middle). It is at this step that the mRNA gets released from the ribosome, because SmpB follows its path and pushes it out of the mRNA exit channel [88]. It has been demonstrated that non-stop mRNA is effectively destroyed and this degradation requires RNase R, as discussed below. Moreover, it has been estimated that RNase R binds to about ~ 1% of translating ribosomes [105], which is very similar to the estimated number of translation reactions undergoing trans-translation (2–4%) [77]. Therefore, it can be assumed that 25–50% of ribosomes undergoing trans-translation are also associated with RNase R, suggesting that the turnover of defective mRNA by RNase R is an extensive mechanism that happens to most of the faulty transcripts.

After the first round of translocation, trans-translation continues like normal translation and the mRNA-like portion of tmRNA is read by canonical translation factors. The tmRNA open reading frame encodes for a small degron sequence that becomes added to the C-terminus of the stalled nascent chain until the included stop codon is reached [106]. Translation subsequently terminates utilizing canonical ribosome release factors [88]. Through canonical ribosome recycling the 70S is then split into subunits and the tagged nascent chain is released. The ribosomal subunits have thus been regenerated and can engage in a new round of translation and the tmRNA-encoded degron induces the removal of the faulty nascent chain by proteases [107].

### Aspects of tmRNA function linked to RNase R

#### Processing and turnover of tmRNA

It has been shown that RNase R participates in tmRNA processing and turnover, although the contribution appears to be minor at normal growth conditions. The genes for RNase R, SmpB and tmRNA (*rnr* – *smpB* – *ssrA*) are part of one operon in *B. subtilis* [70], which suggests that these are co-expressed under conditions that produce transcripts containing the whole set of genes. A similar arrangement of *rnr* and *smpB*, but lacking the gene for tmRNA, is observed in *Streptococcus pneumoniae*. In this organism, this operon appears to be induced in cold-shock with the deletion of RNase R up-regulating the amounts of *smpB* mRNA [67]. In *E. coli, rnr* is not located next to the components of trans-translation, but its expression is also induced by cold-shock [69,108,109]. Therefore, the differences between species could indicate altered requirements for the combined action of RNase R and trans-translation, or it could reflect different strategies by which RNase R activity is regulated, which in *E. coli* has been shown to involve tmRNA-SmpB (see below).

As mentioned, under normal growth conditions tmRNA processing proceeds without a contribution of RNase R, both in *E. coli* [87,110,111] and *B. subtilis* [112]. However, it has been demonstrated in *E. coli* that RNase R can trim tmRNA under cold-shock [108] and that the absence of *rnr* results in defects in trans-translation under this condition [108]. These results therefore indicate a dynamic contribution of RNase R to tmRNA processing that depends on the cellular state. In the alpha-proteobacterium *Caulobacter crescentus*, RNase R does not contribute to tmRNA processing under cold-shock or other conditions, but instead has been shown to degrade it [113]. This could stem from the fact that tmRNA in *C. crescentus* consists of two independent RNA molecules that come together to form a functional complex that carries out trans-translation (this two-part system is conserved in several other bacteria and even organelles) [114,115]. Through this arrangement, a direct entry point for RNase R could be created, that allows the enzyme to participate in tmRNA degradation [113]. Interestingly, it has been shown in *P. syringae* that the deletion of RNase R results in the accumulation of tmRNA fragments after prolonged cold treatment, suggesting that tmRNA turnover in this organism could be initiated by an endonuclease that produces suitable RNA fragments for RNase R-mediated degradation [7]. A further study showed that the absence of RNase R in *P. syringae* results in the upregulation of a type-II toxin-antitoxin system, which could initiate this cleavage and also could produce high levels of non-stop mRNA, both potentially requiring RNase R activity for clean-up (see below) [116]. Strikingly, it has been shown that *E. coli* tmRNA can be cleaved internally by ribotoxins such as RelE [117] and MazF [118] and it has been therefore suggested by Hong and colleagues that RNase R could participate in tmRNA turnover under stress conditions that activate these toxins [113]. Furthermore, it has been demonstrated that SmpB protects tmRNA from degradation *in vitro* [113], which suggests that the main target for degradation would be ‘orphaned’ tmRNA that is not active in trans-translation. Another possible contribution to tmRNA turnover has been described in a *B. subtilis* quadruple deletion strain, lacking RNase PH, RNase R, PNPase and YhaM, which shows accumulation of tmRNA, suggesting a contribution of one ore more of these nucleases to tmRNA degradation [112]. It remains to be seen what the specific contribution of RNase R among those four nucleases is and if the turnover of tmRNA is stimulated under specific growth or stress conditions in *B. subtilis* and other bacteria.

#### Regulation of RNase R expression levels by tmRNA

In *E. coli*, tmRNA controls the amount of RNase R by directly affecting the turnover of RNase R protein at the post-translational level. In *E. coli* RNase R is an unstable protein during exponential growth and becomes stabilized in stationary phase or during cold-shock [109,119]. The stability of RNase R is regulated by the acetylation of one critical lysine residue, K544 in the RNB domain [120]. The acetylation of K544 marks the RNase R protein for degradation by the HslUV and Lon proteases, thereby acting as a regulable degradation signal [119]. It has been demonstrated that the lysine acetylase PatZ is the enzyme that catalyses this reaction and thereby destines RNase R for destruction [120]. PatZ is expressed only during exponential growth phase which explains why RNase R is stabilized in stationary phase [120]. Furthermore, K544 is part of the tri-helix wedge and therefore comes into close contact with the RNA substrate upon catalysis (Figure 1(A)), which can be seen for example in the structure of *M. genitalium* RNase R in complex with ssRNA [19] (PDB identifier 7DIC; The homologous lysine is K550). This suggests that the equivalent K544 in *E. coli* RNase R is protected from acetylation when the enzyme is actively degrading RNA. It has been demonstrated that the components of the trans-translation system directly influence RNase R stability in a ribosome-free form [105], consequently the deletion of either tmRNA or *smpB* increases the *in vivo* half-life of RNase R [121]. Furthermore, Liang and Deutscher have shown that acetylated RNase R is bound strongly by tmRNA-SmpB and that this interaction is necessary for the subsequent recruitment of the HslUV or Lon proteases to the enzyme [119]. The interaction is mediated via the S1-domain and the C-terminal K/R-rich region of RNase R (Figure 1(A,B)) that bind to SmpB [121]. It has been suggested that the acetylation on K544 disrupts a possible interaction of this lysine with the K/R-rich extension, thereby opening up an interaction surface for the binding of SmpB [60], indeed it has been confirmed by small-angle X-ray scattering analysis that the K/R-rich region can fold back onto the RNB domain, supporting this notion [122]. This also suggests that acetylation of K544 could modulate the ability of RNase R to degrade structured substrates, which needs to be tested in the future. In addition, the removal of the S1 domain and/or the K/R-rich extension stabilizes RNase R *in vivo* without significantly altering its RNase activity [121]. Furthermore, it has been shown that the N-terminal HTH-domain of RNase R (Figure 1(A,B)) is necessary for the recruitment of either the HslUV or Lon protease to RNase R [119]. This shows that two unique features of RNase R, the HTH-domain and the K/R-rich region, work together to regulate RNase R expression levels in the cell. Furthermore, a large portion of RNase R is found in a ribosome-bound state and experiments suggest that this protects the enzyme from turnover [105,123,124]. Therefore, it appears that only the free form of the protein is a target for proteolysis [105].

This elaborate control mechanism has so far only been described in *E. coli* and it remains to be studied how RNase R levels are regulated in other organisms. In one study from the Yap lab, that focussed on the analysis of *Staphylococcus aureus*, Lipońska and Yap screened for modifications in the K544 region (*E. coli* numbering) of RNase R to understand if acetylation is conserved in this organism [124]. They show that the equivalent residue to K544 is a glutamine in *S. aureus*, which cannot be acetylated [124]. Importantly, a methylation was detected at a neighbouring residue, which is also a glutamine (Q538, *S. aureus* numbering). This highlights that this region is indeed modified in *S. aureus*, but if the modification contributes to RNase R homoeostasis remains to be studied. The work also showed that RNase R is readily detectable in exponential growth in *S. aureus* which suggests that RNase R levels are not as tightly controlled as in *E. coli* during this growth phase [124]. Interestingly, *B. subtilis*, which is closely related to *S. aureus*, carries a lysine residue at the *E. coli* K544 position, which shows that the *B. subtilis* protein could be acetylated here, which awaits experimental confirmation (Figure 3(B)). Furthermore, RNase R of *P. syringae* lacks a lysine residue in this region, which can possibly contribute to the stable temperature-independent expression of RNase R in this organism [7,8] (Figure 3(B)).

#### Degradation of non-stop mRNAs by RNase R

In *E. coli*, RNase R has been shown to selectively degrade non-stop mRNAs, as well as mRNAs containing rare codons [125]. It has been further demonstrated that this turnover depends on tmRNA and SmpB and that it occurs on stalled 70S ribosomes [125,126]. It has been suggested that RNase R binds to ribosomes in the vicinity of ribosomal protein uS12, which is also engaged by SmpB [88,105,127]. Consequently, in a *smpB* deletion strain, RNase R cannot associate with ribosomes [105] and the ribosome association requires an interaction with the K/R-rich extension of RNase R, similarly to the interaction that leads to protease recruitment [126] (see above). It must be mentioned that the truncation removing the K/R-rich region, studied by Ge et al. is supposed to contain a complete S1 domain (amino acids 1–723 of RNase R), but analysis of a recent AlphaFold model suggests that this truncation also removes the last beta-strand of the S1 domain, likely rendering this domain inactive [25]. Nevertheless, Ge et al. demonstrate the RNase activity of the 1–723 truncation is not impaired, suggesting that it is specifically defective in ribosome association [126]. In a further study, Liang *et al*. confirmed the importance of the K/R-rich extension for ribosome binding and in addition show that the N-terminal HTH-domain of RNase R is not required for this association [105]. The precise binding determinants for RNase R recruitment to stalled ribosomes were described in two follow up studies by the Karzai lab [122,128]. It was shown that the K/R-rich extension is flexible in solution, but adopts a more ordered state upon binding to substrate and that residues E740+K741 and K749+K750, that are located in the K/R-rich region, are key amino-acids for the engagement of RNase R to tmRNA-bound ribosomes [122]. In a second study, Venkataraman and colleagues identified the sequence elements of tmRNA that are required for RNase R engagement [128]. It was found that the last codons of the tmRNA open reading frame play a crucial role in recruiting RNase R to ribosomes [128]. This matches an older study by the same lab where it was shown that the identity of the last three codons in the tmRNA open reading frame is crucial for ensuring efficient non-stop mRNA turnover [129]. Importantly, it was shown in this work that silent mutations to the tmRNA ORF, that do not change the encoded amino acids of tmRNA and do not affect trans-translation activity, still abolish non-stop mRNA decay [129]. Taken together this suggests that the last codons of the tmRNA open reading frame form a structure that can be recognized by RNase R, most likely by the K/R-rich region, and that this is the specific signal that tells the enzyme that stalled ribosomes with non-stop mRNA are present.

Two putative mechanisms for non-stop mRNA decay by RNase R have been previously discussed [128] and we would like to reconcile these two scenarios with the current state of structural and functional information (Figure 3(C–E)):

##### Scenario 1: ‘A-site extraction’

RNase R recognizes the stalled 70S ribosome with accommodated tmRNA by binding the downstream region of the tmRNA open reading frame, most likely with its highly flexible K/R-rich extension (Figure 3(C), left side). (2) The enzyme attaches loosely to the A-site region where uS12 and SmpB are located (Figure 3(D)). (3) The enzyme engages the 3’ end of the truncated mRNA and degrades it, pulling it around SmpB.

This model requires that the bound non-stop mRNA is reachable by RNase R from the A-site (Figure 3(D)). In the accommodated state of tmRNA, the mRNA 3’ end is obscured by SmpB [88,130]. Furthermore, RNase R needs a sufficient single-stranded overhang on its RNA substrate (~7 nucleotides) to begin the turnover reaction. It is therefore questionable if the enzyme could snatch the mRNA 3’ end from the stalled 70S ribosome, even when tmRNA is not present. It is possible that RNase R could act before tmRNA and SmpB have fully settled into the A-site, which would require a precise timing to reach the mRNA first.

##### Scenario 2: ‘Exit channel feeding’

RNase R recognizes the stalled 70S ribosome with accommodated tmRNA by binding the downstream region of the tmRNA open reading frame, most likely with its highly flexible K/R-rich extension (Figure 3(C), right side). (2) The enzyme attaches loosely to the mRNA exit channel (Figure 3(E)). (3) The enzyme samples the mRNA that extrudes from the exit channel through an RNA binding interface (Figure 3(F)). (4) During the first round of tmRNA translocation, the mRNA 3’ end is pushed out of the mRNA exit channel by SmpB (Figure 3(G)). (5) RNase R senses the mRNA 3’ end and captures it (Figure 3(G)). (6) The enzyme engages the 3’ end of the truncated mRNA and degrades it (Figure 3(H)).

In this scenario, RNase R would be directly presented with a single-stranded mRNA that could be sampled to find the 3’ end (Figure 3(F)). There would be no obstruction by SmpB, but one caveat might be that the proposed binding site at the tmRNA ORF might make it hard for the enzyme to reach the mRNA. Nevertheless, we favour this scenario, as it naturally presents RNase R with a 3’ overhang of the mRNA, which is a requirement for the enzyme to act.

In either scenario, RNase R would be released from the ribosome when the tmRNA open reading frame is pulled into the decoding centre and unfolded for translation, which would remove the binding site at the distal part of the tmRNA coding region at the back of the 30S ribosomal subunit (Figure 3(C)).

## rRNA processing by RNase R

The processing of ribosomal RNAs is an important part of ribosome biogenesis and is therefore essential for protein synthesis. An initial study of *E. coli* RNase R showed no accumulation of unprocessed rRNA ends when an *rnr* deletion was combined with a temperature sensitive mutant of PNPase and cells were grown at restrictive temperature [10]. This indicated that RNase R either is not the main factor processing rRNAs in these cells, or that it shares functional overlap with other, highly active, enzymes. A decade later, it was shown by the same group that a quadruple deletion of all 3’ exonucleases in *E. coli* (RNases II, RNase R, RNase PH, and PNPase) results in a large 16S rRNA maturation defect [131], highlighting that any combination of these enzymes could be responsible for its maturation. Furthermore, it was shown that the endonuclease YbeY participates in 16S rRNA maturation in a separate pathway that involves endonucleolytic cleavage at or near the mature 3’ end [132,133]. It was later shown, that RNase R can indeed participate in an YbeY-independent 16S rRNA processing pathway [134,135]. The reaction could be reconstituted *in vitro* by Smith *et al*. using native 17S rRNA-containing biogenesis intermediates and purified RNase R [135]. Further work by the Arraiano lab has implicated RNase R in a functional interaction with the RNA chaperone Hfq, as cells lacking both RNase R and Hfq accumulate high levels of 16S and 23S rRNA-derived fragments [136]. Mirroring what has been shown before, 17S rRNA levels did not change in a *rnr* single deletion mutant indicating that RNase R does not play a major role in 16S rRNA maturation *in vivo* [136].

In *B. subtilis* RNase R has not been shown to participate in rRNA maturation, yet. Importantly, the Condon lab showed that in *B. subtilis* the 3’ end of 16S rRNA is matured by a YbeY homolog, which highlights that this part of ribosome biogenesis is shared across evolution [137]. Furthermore, this work showed that none of the four known 3′-exoribonucleases in *B. subtilis* (RNase R, RNase PH, PNPase and YhaM) play a significant role in the removal of the last remaining 2 nucleotides following YbeY cleavage [137]. Finally, the same work highlighted that RNase R is important for degrading 16S rRNA precursors with elongated 3′ extensions that accumulate once YbeY activity is removed [137]. RNase R therefore contributes to the quality control of 16S rRNA in *B. subtilis* and a further function in 16S rRNA maturation (as shown in *E. coli*) still needs to be elucidated.

Besides the studies in *E. coli* and *B. subtilis*, the contribution of RNase R to rRNA maturation has only been analysed in a few further model systems: A study in *P. syringae* showed that RNase R is required for 5S and 16S rRNA 3′ end maturation in this psychrotrophic gamma-proteobacterium [7] and work in *Arabidopsis thaliana* has shown that an RNase R homolog (RNR1) is required for maturation of all chloroplast rRNAs [15].

## RNase R and rRNA turnover

The number of ribosomes in the cell is an important contributing factor to cell growth and is determined by the equilibrium of ribosome production and ribosome turnover. The destruction of ribosomes by RNases becomes important once the cell is faced with nutrient limitation [138–140], or during acute starvation stress [140–142]. In these cases, the degradation of ribosomes frees up large amounts of cellular resources that enable stress survival, e.g. it has been estimated that turnover of 2000 copies of 23S and 16S rRNA frees enough nucleotides to allow the construction of one bacterial chromosome after conversion to deoxyribonucleotides [141]. Furthermore, damaging agents, like UV-radiation or oxidizing agents can affect both the rRNA and r-protein components of the translation machinery, impeding their function and necessitating their destruction [143]. Most work about rRNA turnover has been performed studying starvation conditions and it has been shown that the amount of RNA is reduced within minutes when an essential nutrient is removed from the growth medium [140]. Nevertheless, a fraction of ribosomes is also degraded during exponential growth [142,144–146] and during the transition from exponential to stationary phase, when large changes to the translation machinery occur that reduce the level of active ribosomes [147–150]. It has been generally proposed that inactive ribosomes, that do not engage in productive translation, will become targets for degradation, because they expose rRNA regions that are otherwise protected in translating 70S ribosomes [142,151]. In 2003, the Deutscher lab showed that RNase R and PNPase work together in rRNA turnover, because the combined inactivation of both enzymes results in a massive accumulation of rRNA fragments, that make up a large portion of RNA in the cell [10]. To study these effects, a heat-sensitive mutant of PNPase was combined with the *rnr* deletion, because the double deletion of *rnr* and *pnp* is inviable [3]. Using this strain, it was shown that rRNA fragments accumulate already after 5 minutes at restrictive temperature and that there is no accumulation of rRNA precursors, indicating that the removal of RNase R and PNPase activities does not affect rRNA maturation [10]. The combined importance of RNase R and PNPase for rRNA turnover has been confirmed by later studies [141,152,153]. A similar effect has been observed when RNase II was inactivated together with PNPase [154], which led to the early suggestion that exonucleases perform a cleanup function that follows initial rRNA fragmentation by endonucleases [155]. Consistently, in *E. coli* it has been reported that the endonuclease RNase E can initially cleave rRNA, supporting this notion [153,156,157]. Furthermore, in *E. coli* a functional overlap in rRNA degradation between RNase R, RNase II and PNPase has been reported, which importantly shows differences during turnover in rRNA quality control that is predominantly handled by RNase R and PNPase and starvation which is realized by RNase R and RNase II [152,153]. The specific involvement of RNase R in the turnover of 16S rRNA during starvation was further confirmed by Prossliner and co-workers, who mapped several 16S rRNA fragments that accumulate in the *rnr* deletion strain [150]. In addition, it has been shown that RNase R works together with the endoribonuclease YbeY to degrade 70S ribosomes containing damaged 30S subunits [133,150].

**Figure 4.**
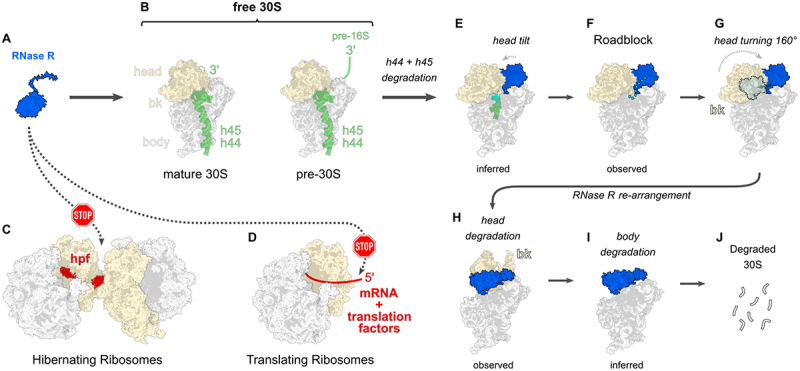
RNase R’s role in the degradation of the small ribosomal subunit. (A) RNase R (blue) engages (B) Free 30S subunits. Both mature and pre-30S can be degraded. (C) Hibernating ribosomes are protected from RNase R action due to steric clashes of RNase R and hibernation factors at the disome interface. In this example hibernation-promoting factor (Hpf) is shown in red. (D) Translating ribosomes are protected from degradation by RNase R through the presence of mRNA and translation factors (indicated in red). (E) First step of 16S rRNA degradation in the context of 30S subunits. RNase R engages the mRNA exit channel region and processively degrades the 16S rRNA starting at the 3’ end. Through this the anti-shine-dalgarno sequence and adjacent rRNA helix 45 and 44 are degraded (comprising the 3’ minor domain of the 30S subunit). (F) RNase R encounters a roadblock once it reaches the neck-to-head transition at helix 28 of 16S rRNA. (G) The 30S subunit head rearranges drastically and RNase R moves with it, anchoring to helix 23 on the 30S body. (H) The roadblock is removed and RNase R can degrade the 30S head, followed by the (I) body until the (J) whole small subunit is degraded.

A specific interaction of RNase R with free 30S ribosomal subunits has been previously reported which is independent from the ribosome interaction that is observed during trans-translation (see above) [123,124,136] (Figure 4(A,B)). It has been shown by the Yap lab, that *S. aureus* RNase R can specifically degrade complete 30S subunits *in vitro*, without engaging 50S subunits [124]. The degradation effect is stronger when ribosomes are isolated from a strain lacking the hibernation-promoting factor (hpf) [124], highlighting the protective function that is conferred by ribosome hibernation [124,150] (for an excellent review on ribosome hibernation see ref [158]. (Figure 4(C)). Importantly, the turnover reaction could be reconstituted without the addition of endoribonucleases, which shows that RNase R by itself is capable of degrading whole ribosomal subunits [124]. In a recent study, we have further analysed the contribution of RNase R to 30S turnover in *B. subtilis* [39]. In this organism RNase R can be purified in a native 30S-bound form using a tagged wild type enzyme, which reveals an enrichment of 30S particles and only small amounts of 70S ribosomes [39]. Using single-particle cryo-EM analysis, we identified a binding location of RNase R at the mRNA exit channel region of the 30S subunit which contains the 3’ end of the 16S rRNA (Figure 4(B)). This result directly explains why hibernating ribosomes are protected from RNase R action due to steric clashes with the Hpf dimer and the ribosome interface of the dimerized 100S ribosome complex (Figure 4(C)). Furthermore, translating ribosomes are expected to be protected from RNase R degradation as well, due to the presence of mRNA and translation factors at the mRNA exit channel [159], which would clash with RNase R upon binding (Figure 4(D)). Therefore, our analysis and the *in vitro* results from Lipońska & Yap indicate that RNase R acts on free 30S subunits [39,124] and that hibernating and translating ribosomes are spared. If the enzyme is capable of degrading the 16S rRNA in the context of 70S ribosomes, as suggested [150], has to be further investigated, but has not been observed by *in vitro* experiments so far [39,124]. Furthermore, it has been shown that biogenesis intermediates of the 30S subunit (pre-30S) can also become targets of RNase R degradation [137]. In principle, RNase R should be able to degrade all pre-30S subunits that present a sufficient 3’ end of the 16S rRNA (Figure 4(B)), which, in turn, might be blocked by biogenesis factors, that cover the 3’ rRNA end [39].

Through cryo-EM analysis, two 30S degradation intermediates could be resolved that contain RNase R, which allowed us to propose a first model of 30S turnover (Figure 4(E–J)): (1) RNase R recognizes the 3’ end of 16S rRNA in a (pre-)30S subunit (Figure 4(B)). (2) Degradation starts from the 3’ end and helix 44 + helix 45 are degraded, RNase R binds to the mRNA exit channel region, which tilts the 30S subunit head (Figure 4(E)). (3) The enzyme hits a roadblock at the transition from the 30S neck region to the head (Figure 4(F)). (4) RNase R induces a massive rearrangement of the whole 30S head and pivots around its HTH-domain that is anchored at the small subunit body (Figure 4(G)). (5) RNase R has overcome the roadblock and can now degrade the 30S head (Figure 4(H)). (6) The reaction proceeds with degradation of the 30S body (Figure 4(I)) until the whole 16S rRNA is degraded (Figure 4(J)).

Our cryo-EM structures reconcile important aspects of the RNase R mechanism discussed above. As shown in Figure 2(C–E), RNase R utilizes a large movement of its accessory domains to stay attached with the 30S subunit while the 30S head turns by ~ 160° (Figure 4(G)). The anchor point of the enzyme is its N-terminal HTH-domain that stays in the same binding location at helix 23 of the 16S rRNA [39]. Importantly, the HTH-domain is a unique feature of RNase R and is not found in its close homolog RNase II (Figure 1(B)) and has not been observed in previous structures of RNase R [19,26]. Similar to the structure of Dis3L2 [36], the new position of the cold-shock domains opens up the access to the tri-helix wedge, that is important for substrate unwinding (shown on the left side of Figure 2(E)). Furthermore, the presence of the cold-shock domains at the neck of the 30S subunit and the fragmented density of both the CSDs and the rRNA in this region (Figure 2(E) (16S rRNA residues beyond A933 are unstructured) could indicate that these domains can assist in destabilizing the ribosomal RNA, resulting in a greater flexibility of the 30S head, before it is turned (Figure 4(G)).

Finally, no cryo-EM density for the C-terminal K/R-rich region is observed in the 30S turnover states [39], indicating that this unique extension of RNase R does not play a universal role in ribosome binding and instead might represent an adaptation to specific processes, like trans-translation [122].

## Concluding remarks

Throughout evolution, living cells have used RNases to regulate the large number of coding and non-coding RNAs present in each organism. It has been demonstrated that several RNase families are universally distributed in bacteria [1] and we are learning more and more about the remarkable conservation of catalytic mechanisms, but also about specific adaptations to the physiological niche of each family. Most work about RNase R has been performed using *E. coli* as a model organism, which has limited our understanding of the full evolutionary picture of RNase R biology. Some previous studies have already shown that profound differences in RNase R functions exist in other species: For example, the divergent regulation of protein levels in *S. aureus* [124], a strongly increased sensitivity to substrate methylation in *M. genitalium* [19], or the remarkable temperature-dependent switching in the ability to degrade RNA structures in *H. volcanii* [14]. These examples highlight, that much can be learned from studying the whole evolutionary picture of RNase R which could yield new technological improvements (e.g. modification-sensitive enzymes for omics research), or medical interventions targeting specific pathogenic variants of this important enzyme.

We believe that the following points will be of special interest for future studies:

### Regulation of RNase R protein levels and activity

Because RNase R is a potent enzyme which degrades nearly all RNAs (that is all RNAs with a sufficient 3’ overhang), its activity needs to be carefully controlled. It has already been shown that *E. coli* possesses an elaborate regulation mechanism, that utilizes lysine acetylation [120] and direct binding partners to control RNase R protein levels.

Future questions: How do other organisms regulate RNase R activity? How do they protect their ribosomes from RNase R action?

### Sensitivity towards nucleotide modifications and RNA lesions

Native RNA substrates can contain a huge variety of modifications or even damage-induced RNA lesions (e.g. 8-oxo-G as a result of oxidative stress). Most *in vitro* studies on RNase R have used short and synthetic RNA substrates without modifications. Therefore, we are missing a large piece of information of how RNase R reacts to various native RNA modifications. Furthermore, we already know that RNase R homologs react differently to RNA 2’-O-methylation [19].

Future questions: Which modifications stall RNase R? Which RNA lesions are still tolerable? Can we develop the enzyme to be sensitive to specific modifications?

### Interplay with other RNA decay factors or helicases

RNase R is not the only RNA decay enzyme in the cell and as such synergistic effects with other RNases are assumed. For example, an interaction of RNase R with RNase E has been proposed in *P. syringae* [160]. Furthermore, it has been demonstrated that several endonucleases (e.g. RNase E and YbeY [137,152,156] can produce substrates for RNase R.

Future questions: Are there more endonuclease or helicase partners that engage with RNase R in specific cellular functions? Does RNase R form larger RNA decay complexes through defined protein–protein interactions?

### Participation of RNase R in non-stop mRNA degradation

Studies have shown that RNase R greatly contributes to tmRNA-dependent mRNA degradation on stalled ribosomes and we have learned many contributing factors to this process in *E. coli*.

Future questions: Does RNase R contribute to non-stop mRNA decay across evolution? What is the exact mechanism of tmRNA-dependent non-stop mRNA decay in *E. coli* and other organisms? Is the turnover reaction of non-stop mRNA connected to RNase R-mediated 30S subunit degradation?

These and many other questions will be elucidated by continued research into this important nuclease which will, without doubt, provide many exiting findings that will inform our understanding of basic bacterial physiology and evolution, as well as contribute to technical and medical applications.

## Data Availability

No data was generated or analysed for this work.
